# Supplementary material for: Causal Relationship between Aging and Anorexia Nervosa: A White-Matter-Microstructure-Mediated Mendelian Randomization Analysis
Source: Biomedicines. 2024 Aug 16;12(8):1874. doi: 10.3390/biomedicines12081874 (PMC11351342; doi:10.3390/biomedicines12081874)
Supplement: Supplementary file 1 [file biomedicines-12-01874-s001.zip › Supplementary Figure/Figure S4/Singe SNP plot of MR analysis for TL to ubm-b-1523.pdf]

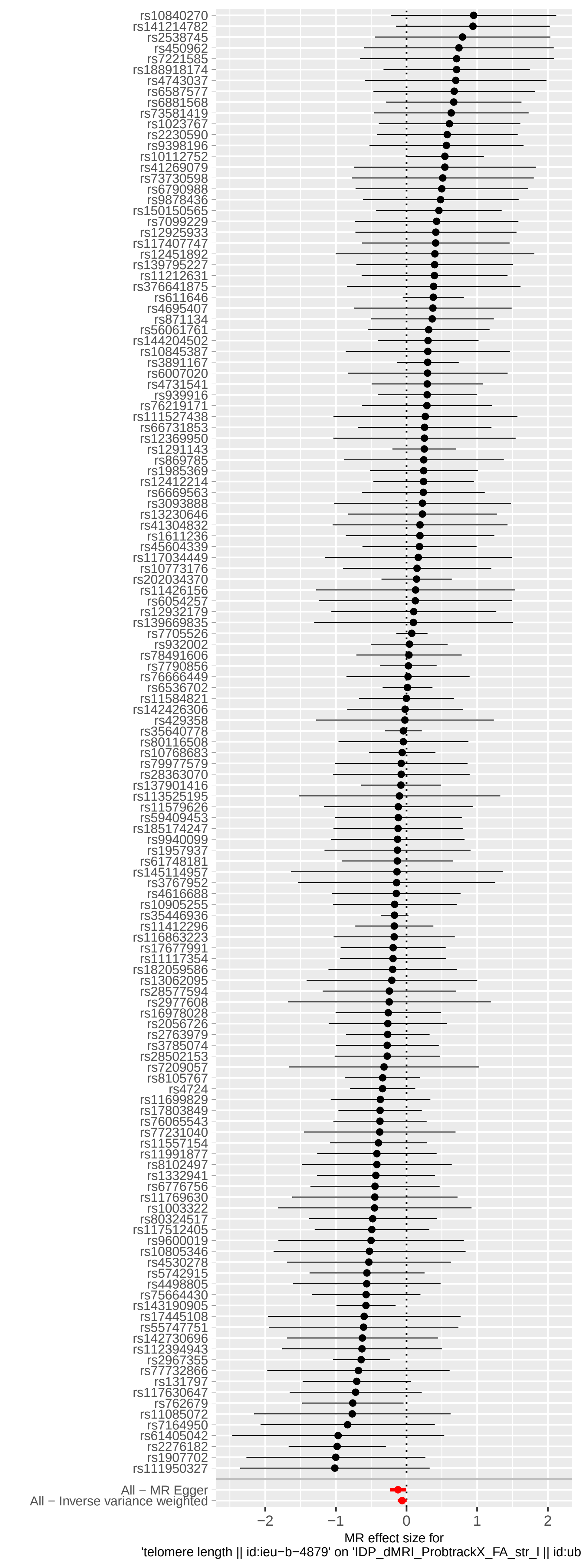
rs10840270
rs141214782
rs2538745
rs450962
rs7221585
rs188918174
rs4743037
rs6587577
rs6881568
rs73581419
rs1023767
rs2230590
rs9398196
rs10112752
rs41269079
rs73730598
rs6790988
rs9878436
rs150150565
rs7099229
rs12925933
rs117407747
rs12451892
rs139795227
rs11212631
rs376641875
rs611646
rs4695407
rs871134
rs56061761
rs144204502
rs10845387
rs3891167
rs6007020
rs4731541
rs939916
rs76219171
rs111527438
rs66731853
rs12369950
rs1291143
rs869785
rs1985369
rs12412214
rs6669563
rs3093888
rs13230646
rs41304832
rs1611236
rs45604339
rs117034449
rs10773176
rs202034370
rs11426156
rs6054257
rs12932179
rs139669835
rs7705526
rs932002
rs78491606
rs7790856
rs76666449
rs6536702
rs11584821
rs142426306
rs429358
rs35640778
rs80116508
rs10768683
rs79977579
rs28363070
rs137901416
rs113525195
rs11579626
rs59409453
rs185174247
rs9940099
rs1957937
rs61748181
rs145114957
rs3767952
rs4616688
rs10905255
rs35446936
rs11412296
rs116863223
rs17677991
rs11117354
rs182059586
rs13062095
rs28577594
rs2977608
rs16978028
rs2056726
rs2763979
rs3785074
rs28502153
rs7209057
rs8105767
rs4724
rs11699829
rs17803849
rs76065543
rs77231040
rs11557154
rs11991877
rs8102497
rs1332941
rs6776756
rs11769630
rs1003322
rs80324517
rs117512405
rs9600019
rs10805346
rs4530278
rs5742915
rs4498805
rs75664430
rs143190905
rs17445108
rs55747751
rs142730696
rs112394943
rs2967355
rs77732866
rs131797
rs117630647
rs762679
rs11085072
rs7164950
rs61405042
rs2276182
rs1907702
rs111950327
All − MR Egger
All − Inverse variance weighted
−2
−1
0
1
2
MR effect size for
'telomere length || id:ieu−b−4879' on 'IDP_dMRI_ProbtrackX_FA_str_l || id:ub
